# Optimisation of Ionic Models to Fit Tissue Action Potentials: Application to 3D Atrial Modelling

**DOI:** 10.1155/2013/951234

**Published:** 2013-07-01

**Authors:** Amr Al Abed, Tianruo Guo, Nigel H. Lovell, Socrates Dokos

**Affiliations:** Graduate School of Biomedical Engineering, The University of New South Wales, Sydney, NSW 2052, Australia

## Abstract

A 3D model of atrial electrical activity has been developed with spatially heterogeneous electrophysiological properties. The atrial geometry, reconstructed from the male Visible Human dataset, included gross anatomical features such as the central and peripheral sinoatrial node (SAN), intra-atrial connections, pulmonary veins, inferior and superior vena cava, and the coronary sinus. Membrane potentials of myocytes from spontaneously active or electrically paced *in vitro* rabbit cardiac tissue preparations were recorded using intracellular glass microelectrodes. Action potentials of central and peripheral SAN, right and left atrial, and pulmonary vein myocytes were each fitted using a generic ionic model having three phenomenological ionic current components: one time-dependent inward, one time-dependent outward, and one leakage current. To bridge the gap between the single-cell ionic models and the gross electrical behaviour of the 3D whole-atrial model, a simplified 2D tissue disc with heterogeneous regions was optimised to arrive at parameters for each cell type under electrotonic load. Parameters were then incorporated into the 3D atrial model, which as a result exhibited a spontaneously active SAN able to rhythmically excite the atria. The tissue-based optimisation of ionic models and the modelling process outlined are generic and applicable to image-based computer reconstruction and simulation of excitable tissue.

## 1. Introduction 

Mathematical models have been valuable tools in the field of electrophysiology, providing quantitative insights of natural processes. The majority of these models are generic in a sense that they describe a biological phenomena documented over a number of observations. However, sometimes the interspecimen variability is important *per se* in understanding the mechanisms underlying a biological process and/or how it is modulated by pathological, pharmacological, or environmental factors. For such studies, it is advantageous to develop subject-specific biological models for each particular case investigated. Generic quantitative conclusions can be then drawn from a family of subject-specific models. However, as in nature, subject-specific models should not be developed in isolation but be able to operate within a larger encompassing biological context (a higher scale of modelling hierarchy in physiome terminology [1]) and still produce useful predictions. The influence of the surrounding environment on the behaviour of each subject should be built into the subject-specific models. In this study a methodology for subject-specific modelling is presented, using cardiac atrial electrophysiology as a basis. 

Atrial fibrillation (AF) is the most common form of arrhythmia in the clinic, estimated in 1997 to affect 2.2 and 4.5 million people in the USA and EU, respectively [2]. It is most prevalent among the elderly, affecting approximately 8% of people over 80 years of age and is associated with changes to the structure of the atria and a major indicator of stroke [2]. A number of pharmacological and surgical approaches have been used to control atrial arrhythmias. As the efficacy of these interventions is not very high, subject-specific computational models are useful to better understand underlying mechanisms initiating and maintaining the arrhythmia and assess the appropriate interventions.

Computer simulations of cardiac electrophysiology are based on single-cell ionic models, which can be incorporated into tissue or whole-heart simulations. Over the last decade or so, with the advance of and reduced costs of computational resources, there has been a proliferation of 3D morphologically realistic electro-anatomical models of the human atria (e.g., [3–7]).

The single-cell ionic models are either phenomenological, able to explicitly generate action potential (AP) waveforms, or, based on equations describing the detailed gating kinetics of various ion channels, exchangers and transporters in the cell's membrane and intracellular compartments. In recent years, a number of groups have used various automated algorithms to optimise the parameter values and fit ionic models to experimentally recorded APs. A curvilinear gradient method [8] was used to fit the Beeler and Reuter model [9] to a model-generated ventricular AP [10]. Syed et al. [11] used a genetic algorithm to fit the Nygren et al. [12] human atrial cell model to experimental and model-generated AP waveforms obtained from an alternate atrial cell ionic model [13]. A particle swarm algorithm was used to fit the 4-variable Cherry et al. [14] model to model-generated human atrial APs [15]. Syed et al. [11] suggested that the use of a more realistic pulse to stimulate the ionic model produced improved AP waveform fits. This idea was further improved by optimising the AP from a single point in a 1D ring model of electric propagation, to take into account electrotonic interactions during excitation and propagation [16]. However, the goodness of the fit was only verified by comparing the values of the fitted and original parameters, rather than the AP morphologies.

A naive implementation of parameters from single-cell ionic models into higher-order geometries might not reproduce expected propagation or activation patterns. For example, Garny et al. [17] reported that the default parameters of the Zhang et al. [18] central and peripheral sinoatrial node (SAN) cell models needed to be modified so that the SAN could generate spontaneous firing in a 1D cable model. In addition, they had to increase the intercellular conductivity for SAN and atrial regions to ensure that the central SAN, as opposed to the periphery, was the leading pacemaker site [17]. Alternatively, it is possible in higher dimensional models to adjust tissue conductivity and ion channel density gradients to produce rhythmic spontaneous SAN activation and physiological atrial excitation [19]. 

Overcoming such issues necessitates a parameter optimisation approach which is able to account for the electrotonic interactions between regions of different electrophysiological properties while maintaining an optimal fit of model APs to whole-tissue experimental data. In this study a process was developed whereby parameters of a generic single-cell ionic model were estimated by fitting APs generated by heterogeneous 2D disc tissue models to APs recorded experimentally from whole-tissue preparations. This approach allows the cell models to preserve their individual properties and yet operate under the electrotonic influence of nearby cells with different properties. The resulting optimised parameters were then implemented in a 3D human atrial geometry to produce spontaneous SAN activation and excitation of the atria. The electrically heterogeneous single-cell ionic properties are integrated into a larger biological tissue and collectively give rise to higher-order behaviour, including propagation in the atria. 

## 2. Methods 

### 2.1. *In Vitro* Electrophysiology

All experimental procedures were conducted in accordance with the Australian National Health and Medical Research Council Guidelines and were approved by the University of New South Wales Animal Ethics and Care Committee.

New Zealand white rabbits of both sexes (age 6–24 months) were anaesthetised with isoflurane (5% in O_2_), and heparin (1000 IU) was administered intravenously into the marginal ear vein. Subsequently, a thoracotomy was performed, cold cardioplegia solution (4°C) of the following composition (mM) 110 NaCl, 16 KCl, 16 MgCl_2_·6H_2_O, 1.2 CaCl_2_·2H_2_O was poured into the chest cavity, and the heart was excised by rapidly sectioning the great vessels. The heart was immersed in the above Cardioplegia solution to clean away any remaining blood. It was then mounted in a dissection chamber and superfused with Tyrode's solution of the following composition (mM) 130 NaCl, 4 KCl, 1.2 CaCl_2_, 0.5 MgCl_2_, 1.8 NaH_2_PO_4_, 18 NaHCO_3_, 10 glucose. This solution was gassed with carbogen (95% O_2_ and 5% CO_2_) to maintain the pH at ~7.4 and regularly exchanged with fresh Tyrode's solution during the dissection procedure. Depending on the experiment, SAN/right atrial (SAN/RA) or left atrial/pulmonary vein (LA/PV) tissue preparations were dissected. 

Each preparation was then mounted using fine entomological pins, endocardial surface up to the Sylgard floor of a modified RC-26 recording chamber (Warner Instruments, USA) and continuously superfused at 4 mL·min^−1^ with Tyrode's solution using two MP-II Mini peristaltic pumps (Harvard Apparatus, USA). The temperature of the bathing solution was maintained at 32 ± 1°C using a feedback regulated temperature controller (TC-324B, Warner Instruments, USA) which heated the recording chamber, as well as a custom heater which preheated the solution before it entered the chamber. The tissue was allowed to recover from surgical trauma for approximately one hour before microelectrode recording commenced. 

Intracellular glass microelectrodes (resistance 50–100 MΩ), pulled using a P97 Sutter puller (Sutter Instruments, USA) and filled with 3 M KCl, were used to impale tissue-intact myocytes within the tissue preparation and record intracellular potentials relative to a Ag/AgCl pellet immersed in the bathing solution. The glass microelectrode was connected to the headstage of an Axoclamp 2B amplifier (Axon Instruments/Molecular Devices, USA) which was used to amplify (gain ×10) and filter (low-pass cutoff of 10 kHz) the membrane potential. The output of the Axoclamp amplifier was digitised using a USB-6009 or USB-6251 data acquisition device at sampling rate of 20 kHz (National Instruments, USA), run using a custom LabVIEW (National Instruments, USA) software interface. The stimulus current was recorded on a second channel simultaneously with the membrane potential. 

Spontaneous electrical activity was recorded from the SAN/RA tissue preparations, but the LA/PV preparations required an external pacing stimulus. Monophasic suprathreshold pulses (2 ms duration and 100–800 *μ*A in amplitude) from an STG1002 isolated constant-current stimulator (MultiChannel Systems, Germany) were used to stimulate the latter tissue preparation using a pair of bipolar stainless steel electrodes (125 *μ*m in diameter) coated with Teflon with tips exposed. 20–40 *μ*M of blebbistatin (Toronto Research Chemicals, Canada), an excitation-contraction decoupler, was added to the recirculating superfusate solution for the LA/PV recordings.

### 2.2. Generic Ionic Model Development

**Figure 1:**
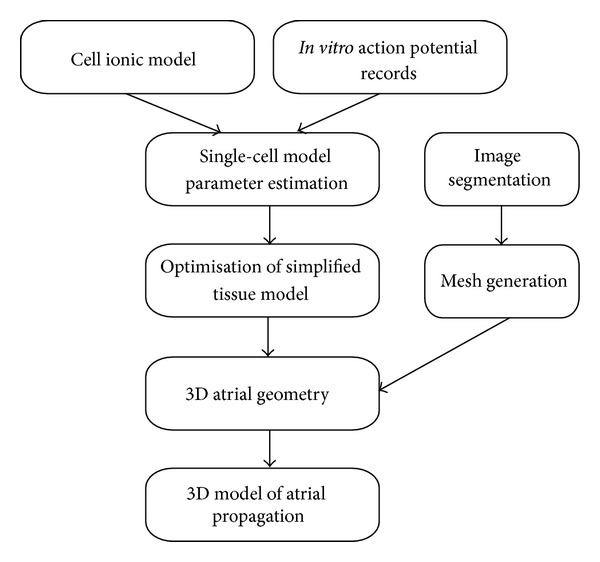
Outline of protocol followed for developing the 3D atrial model with heterogeneous regions and optimised generic model parameters.

**Figure 2:**
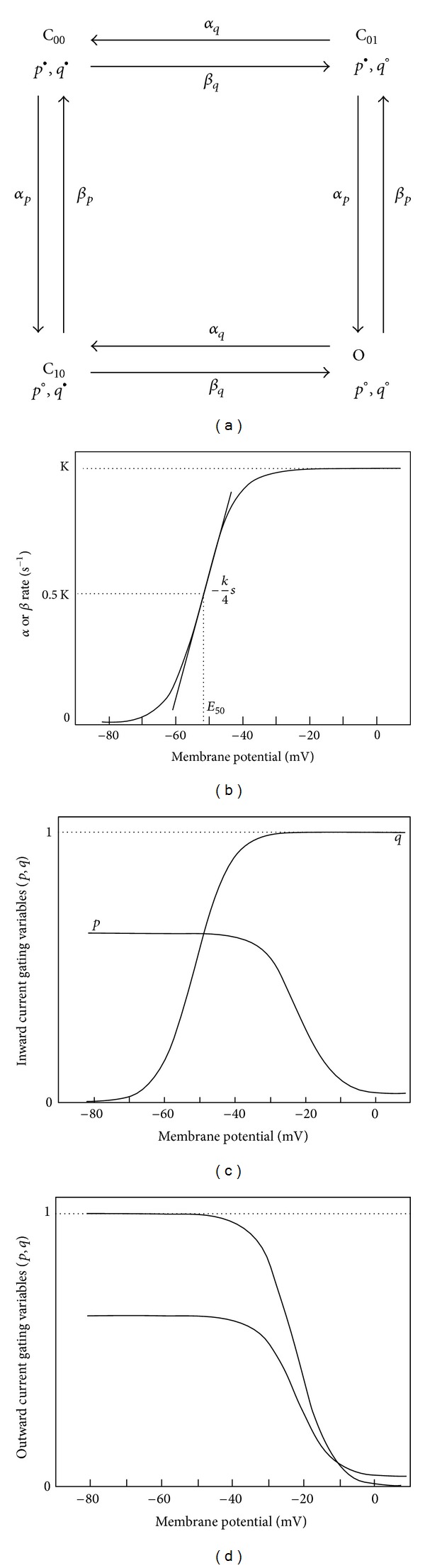
Generic ionic model structure. (a) Equivalent Markov state diagram of ionic currents. O represents the open or conducting state for the ionic channel, and C_00_, C_01_, and C_10_ are the closed states. *p* and *q* are two gating variables governing the kinetics. ○ Open gate, ● Closed gate. (b) Typical profile of rate variable (*α* or *β*) as a function of membrane potential. Each rate is defined by three parameters: *k*, a maximum plateau value, *E*_50_, the membrane potential at half the maximum value, and *s*, which is related to the slope at the *E*_50_, such that $\overset{´}{\alpha }\left(E_{50}\right)=-ks_{\alpha }/4$ and $\overset{´}{\beta }\left(E_{50}\right)=-ks_{\beta }/4$. (c) Typical steady-state profile for *p* and *q* as a function of transmembrane potential for a transiently activating inward current. (d) Typical steady-state profile of *p* and *q* for a sustained outward current, as a function of transmembrane potential.

The first step in the proposed modelling process (Figure 1) is to model the behaviour of the fundamental element of the biological system under study: namely, the atrial cardiomyocytes. It is important for the ionic models describing cellular electrical activity to be generic, so that they can be adapted and applied to heterogeneous myocytes of the tissue in question. A generic cell ionic model was formulated based on standard template equations for each ionic current, including gating and rate variables (Figure 2). 

The governing equation for the change in membrane potential is given by

(1) $$\begin{array}{c} \frac{dE_{m}}{dt}=\frac{-1}{C_{m}}\left(I_{L}+\sum_{j=1}^{N}I_{j}\right), \end{array}$$

where *E*_*m*_ (mV) is the membrane potential, *I*_*j*_ (nA·cm^−2^) and *C*_*m*_ (*μ*F·cm^−2^) are the total ionic current and capacitance per unit cell membrane area, respectively. *C*_*m*_ was fixed to 1 *μ*F·cm^−2^ for all models. The ability to reproduce complex dynamic behaviours in membrane potential in response to various experimental manipulations is provided for allowing a user-specified number of time-dependent ionic currents (*N*) in (1). Selection of the appropriate value of *N* is dependent on the level of detail the model is required to capture. In this study, our aim was to reproduce APs recorded from spontaneously active intact myocytes or quiescent myocytes externally paced using a single stimulation frequency and therefore only two active currents (*N* = 2) were used (a general inward and a general outward) in addition to a leakage current.

The time-independent leakage current (*I*_*L*_) in (1) was described by

(2) $$\begin{array}{c} I_{L}={\overset{-}{g}}_{L}\left(E_{m}-E_{\text{rev},L}\right), \end{array}$$

where $\;{\overset{-}{g}}_{L}\;$ (*μ*S·cm^−2^) is the maximum conductance, *E*_rev,*L*_ (mV) the reversal potential. Similarly, each generic time-dependent current (*I*_*j*_) in (1) was given by

(3) Ij=g−j pj qj(Em−Erev,j),dpjdt=αp,j(1−pj)−βp,jpj,dqjdt=αq,j(1−qj)−βq,jqj,

where $\;{\overset{-}{g}}_{j}$ (*μ*S·cm^−2^) is the maximum conductance, *E*_rev,*j*_ (mV) the reversal potential, and *p*_*j*_ and *q*_*j*_ are gating variables for the *j*th active ionic current in (1). The steady-state values of *p* and *q* are given by

(4) $$\begin{array}{c} p_{\infty j}=\frac{\alpha_{pj}}{\left(\alpha_{pj}+\beta_{pj}\right)},\\ q_{\infty j}=\frac{\alpha_{qj}}{\left(\alpha_{qj}+\beta_{qj}\right)}. \end{array}$$

Example plots of these steady-state profiles are shown in Figures 2(c) and 2(d). *α* and *β* in (4) are forward and reserve rates (with units of s^−1^) for each gating variable, each a function of *E*_*m*_ only, and determined by

(5) $$\begin{array}{c} \alpha =\frac{k_{\alpha }}{1+e^{s_{\alpha }(E_{m}-E_{50})}}, \end{array}$$

(6) $$\begin{array}{c} \beta =\frac{k_{\beta }}{1+e^{s_{\beta }(E_{m}-E_{50})}}, \end{array}$$

where *k*_*α*_, *k*_*β*_ (s^−1^), *E*_50_ (mV), *s*_*α*_, and *s*_*β*_ (mV^−1^) are rate-dependent variables for each gate (*p*_*j*_, *q*_*j*_) of *I*_*j*_.

### 2.3. Ionic Model Parameter Optimisation

The parameters of the generic ionic model given by (1)–(6) were automatically adjusted to reproduce cardiac AP waveforms recorded experimentally from SAN, RA, LA, and PV tissue-intact myocytes. Parameters were optimised in two stages. Initially it was assumed that the myocytes behaved in isolation of neighbouring elements in accordance with (1). Thus AP waveforms generated by the single-cell ionic model were first fitted to experimentally recorded APs to arrive at a set of optimised initial parameter values. In the subsequent stage, model parameters were further optimised in the presence of electrotonic loading from neighbouring myocytes with heterogeneous electrophysiological properties.

In both stages, a custom curvilinear gradient optimisation method was used to fit the model to experimentally recorded AP waveforms by minimising a weighted objective function [8, 10]. For each optimisation run, the weight function was initially user specified but then dynamically updated according to the residuals between experimental and model data points. In addition, a set of upper/lower constraints were imposed on each model parameter (Table 1). The constraints on *E*_rev,*j*_ determined whether the *j*th time-dependent current was inward or outward. For example when optimising the model to fit cSAN APs, the reversal potential of currents 1 and 2 was constrained to be in the [−100.0 − 70.0] mV and [5.0  100.0] mV ranges to define an outward and an inward current, respectively. Moreover the values for *s*_*α*_ and *s*_*β*_ of the *p* and *q* gates of each outward current shared the same range, whereas those for the *p* and *q* gates of each inward current had opposite signs. Hence the steady-state *p* and *q* curves for each inward current exhibited opposing trends (with respect to membrane voltage), whilst those of each outward current exhibited the same trend (Figure 2).

### 2.4. Optimisation of 2D Tissue Disc Models

**Figure 3:**
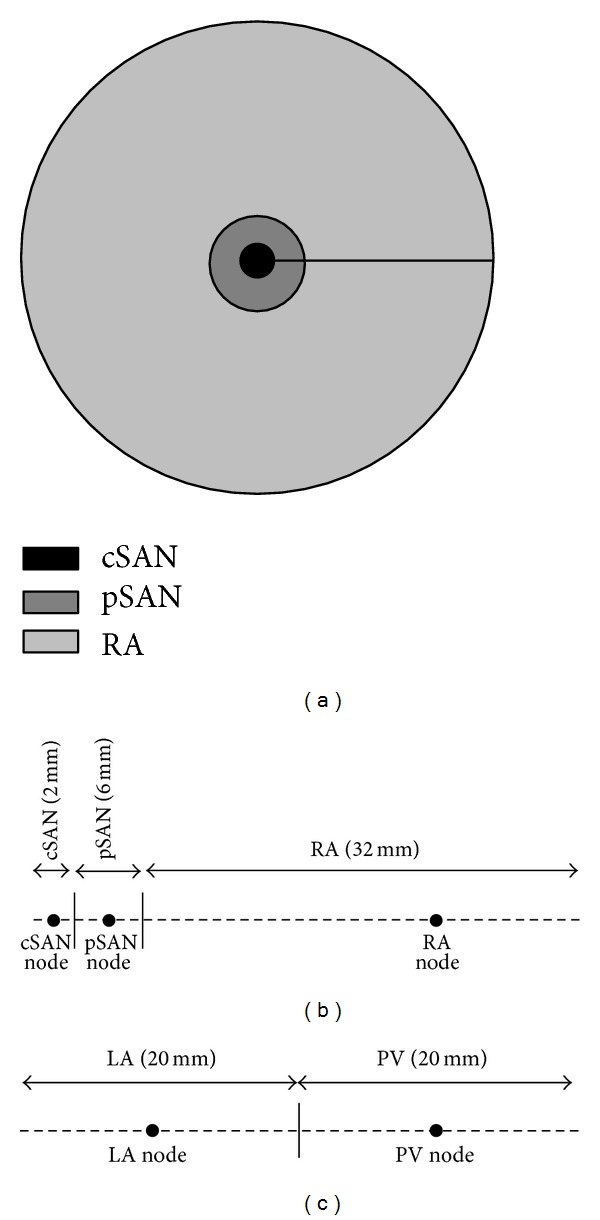
Setup of 2D disc models. (a) 2D disc approximation of the human SAN and surrounding atrial tissue. (b) 1D cable representation of 2D axisymmetric disc. AP waveforms at shown selected nodes representing cSAN, pSAN, and atrial tissue were optimised. (c) 1D cable representation of an LA-PV disc.

In order to better account for the electrotonic interaction between myocytes, a 1D numerical approximation of an axisymmetric heterogeneous 2D disc model of electrical propagation was developed based on the generic ionic model described above and a modified form of the cable equation [20]

(7) $$\begin{array}{c} \frac{\partial }{\partial r}\left(\frac{r\sigma b}{2}\frac{\partial E_{m}}{\partial r}\right)=rI_{m}, \end{array}$$

where *σ* (*μ*S·cm^−1^) is the tissue conductivity, *r* (cm) is the distance from the centre of the disc, *b* = 2 × 10^−3^ (cm) is the disc thickness, assuming it is one cell layer thick, and *I*_*m*_ (nA·cm^−2^) is the total membrane current, comprised of capacitive and ionic components according to

(8) $$\begin{array}{c} I_{m}=C_{m}\frac{dE_{m}}{dt}+I_{L}+\sum_{j=1}^{N}I_{j}, \end{array}$$

where all the variables are as defined in (1). To represent myocyte heterogeneity in the atrium, the cable was divided into the appropriate number of sections, each with its distinct tissue conductivity value and ionic model parameters. Two axisymmetric discs were considered: one representing a right atrial preparation comprised of a central SAN (cSAN) region, a peripheral SAN (pSAN) region, and surrounding right atrium (RA), and the other representing a left atrial preparation comprised of left atrium (LA) and a pulmonary vein (PV) region. These two disc models will be referred to as cSAN-pSAN-RA and LA-PV, respectively (Figure 3). The tissue conductivity was selected to produce known conduction velocity (CV) in each segment: 20–30 cm·s^−1^ in the central and peripheral regions of the SAN and 80–100 cm·s^−1^ in the right and left atria. APs generated at selected points from each section of the cable were fitted to APs recorded experimentally from the corresponding intact myocyte by optimising the ionic model parameters assigned to that particular section of the disc. 

The equation was solved using the method of lines (spatial derivatives calculated from finite differences) implemented in MATLAB (MathWorks, USA) using the ODE15s solver (absolute tolerance = 10^−6^, relative tolerance = 10^−3^, using an adaptive time stepping routine with a maximum time step of 2 ms). The 4 cm cable was discretised into 21 equi-spaced nodes (internodal distance = 2 mm).

The tissue conductivity of the first atrial node in the cSAN-pSAN-RA cable was reduced to equal the conductivity of both SAN regions. In the case of the LA-PV axisymmetric disc, a suprathreshold stimulus pulse of 2 ms duration was applied to excite the first node at the centre of the disc, generating an impulse that propagated from the LA to PV segments. 

### 2.5. 3D Atrial Model

The final stage in the modelling process (Figure 1) is to incorporate models of individual elements of the biological system, each optimised to function in the presence of other elements, into a realistic environment and simulate the behaviour of the entire system. 

The generic ionic model parameters for cSAN, pSAN, RA, LA, and PV myocytes obtained from the above 2D axisymmetric model's optimisation were integrated into a 3D atrial model. The 3D atrial geometry was reconstructed from cryosection images of the male Visible Human obtained from the US National Library of Health [21]. The images had a resolution of 0.33 mm × 0.33 mm and were axially spaced at 1.0 mm intervals. The raw slices were registered along the *z*-axis and converted to a 24-bit greyscale format using MATLAB. The images were then imported to ScanIP (Simpleware Ltd., Exeter, UK) for segmentation into different masks using a combination of floodfill algorithms, Gaussian recursion, and morphological and binarisation filters, in addition to manual segmentation and cleanup. 

**Figure 4:**
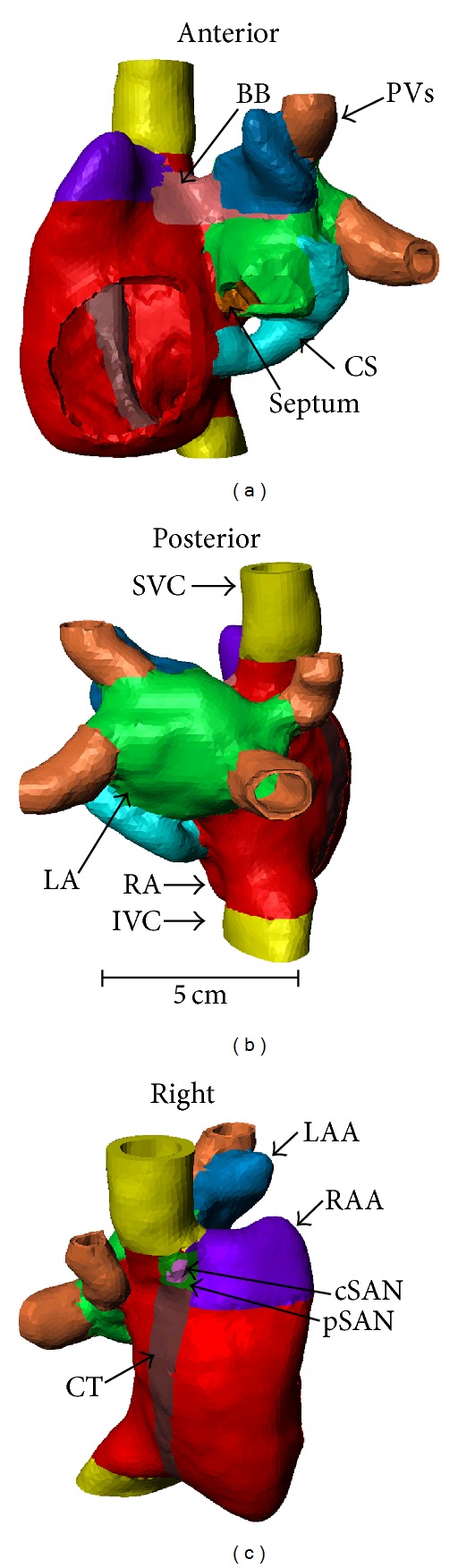
Different views of 3D atrial geometry, with segmented regions highlighted in different colours. BB: Bachmann's bundle, CS: coronary sinus, PV: pulmonary vein, LA: left atrium, RA: right atrium, LAA: left atrial appendage, RAA: right atrial appendage, CT: crista terminalis, cSAN: central sino-atrial node, pSAN: peripheral sino-atrial node, SVC: superior vena cava, and IVC: inferior vena cava.

The greyscale images were segmented into twelve distinct regions thought to be important in electrical conduction, in genesis or maintenance of reentrant wavefronts, or as sites of ectopic foci (Figure 4). In addition to RA and LA domains, the crista terminalis (CT), right and left atrial appendages (RAA, LAA), and superior and inferior venae cava (SVC, IVC) were also segmented. Intra-atrial domains included the septum, Bachmann's bundle (BB), and coronary sinus (CS). Preliminary segmentation revealed what appeared to be additional right and left PVs. These were manually removed at their intersection with the LA chamber. Histological studies have shown that cardiomyocytes can extend up to 20 mm into the PV walls as myocardial sleeves [22]. The four PVs were cropped proximally to their first branching point, or at a maximum length of 20 mm. The SAN domain was manually determined using anatomical landmarks and then further subdivided into cSAN and pSAN regions taking care to ensure that all cSAN voxels had contact with pSAN but not atrial voxels. The dimensions of the resultant SAN domain (13 mm in length and 7 mm in width) and its distance to the endocardial surface of the atria were within the range of values reported histologically [23]. Finally, all domains were individually filtered using a morphological filter and/or smoothed against each other as appropriate and then downsampled to a resolution of 2 mm × 2 mm × 2 mm. 

**Figure 5:**
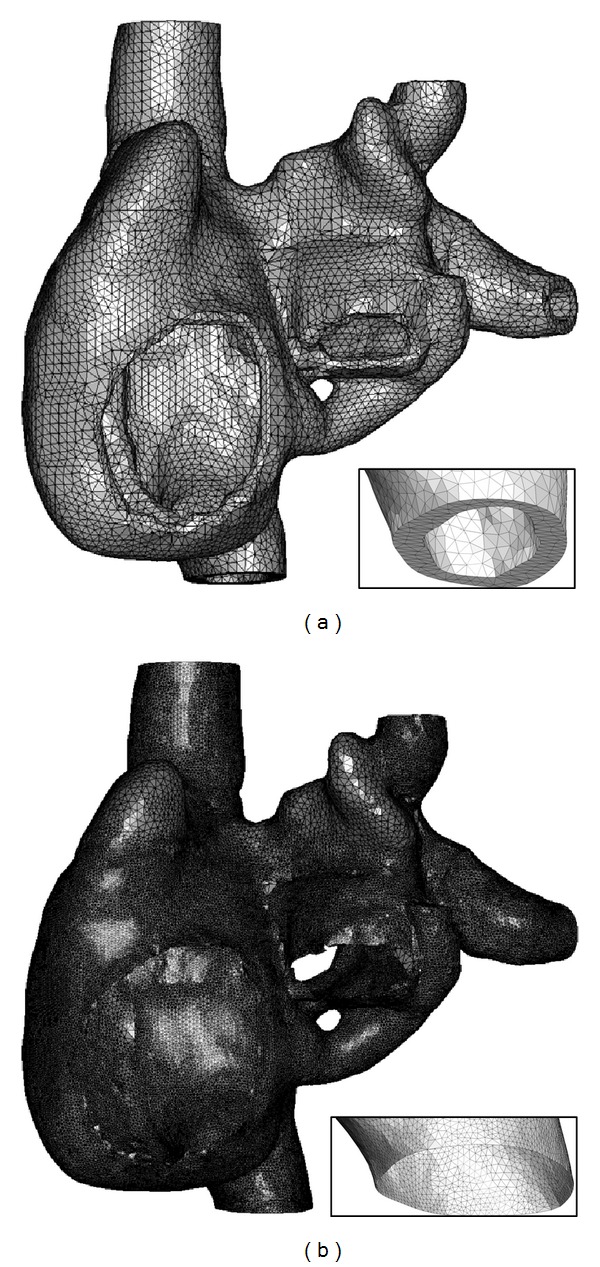
3D atrial finite element meshes. Anterior and posterior views of the (a) coarse and (b) refined atrial meshes. (a) illustrates the 3D realistic version of the atria with finite wall thickness, whilst (b) illustrates the 2D shell surface version. Insets: zoomed views of the inferior vena cava (IVC) highlighting the 3D finite wall and shell wall structures, respectively.

Simpleware's +FE free algorithm was used to generate either a coarse tetrahedral mesh with finite atrial wall thickness or a refined epicardial surface triangular mesh with all domains meshed simultaneously to ensure proper contact areas (Figure 5). Mean element sizes were 3.388 mm and 0.991 mm for the finite wall and surface meshes, respectively. These sizes were calculated based on the longest edge of each element. A mesh refinement analysis was undertaken to validate the effect of mesh element size on electric propagation in representative geometries. There was an approximately 10% decrease in CV in a 2D rectangular model, and no change in CV in the 3D cube model as the mesh element size was reduced from 3.5 mm and 2.0 mm to 246 *μ*m in the respective geometries. 

Atrial propagation was modelled using the monodomain formulation

(9) $$\begin{array}{c} \nabla \cdot \left(\sigma \nabla E_{m}\right)=A\left(I_{m}+I_{b}\right), \end{array}$$

where *A* (cm^−1^) is the surface to volume ratio of the myocyte, *σ* (*μ*S·cm^−1^) is the tissue conductivity, and *I*_*m*_ (nA·cm^−2^) is the total membrane current given by (1). A constant background hyperpolarizing current (*I*_*b*_) of 79 nA·cm^−2^ or 160 nA·cm^−2^ was applied to the cSAN and pSAN regions of the finite wall or epicardial shell atrial models, respectively, to slow the rate of spontaneous pacemaking activity of the optimised rabbit cSAN and pSAN generic ionic models to a human baseline heart rate of 77 and 75 pulses per minute for the respective models. 

Each electrophysiological region was assigned one set of ionic parameters previously obtained using the 2D axisymmetric disc optimisation (see Tables 2 and 3 for a list of initial variable and parameter values). Optimised RA parameters were assigned to the RA, RAA, septum, SVC, IVC, and CT regions. Optimised LA parameters were assigned to the LA, LAA, and BB regions. Different values of tissue conductivity were assigned to distinct regions based on known conduction velocities: atrial bulk tissue (RA, RAA, LA, LAA, and septum, CV *≈* 80 cm·s^−1^), fast conduction regions (CT and BB, CV *≈* 150 cm·s^−1^) and slow conduction regions (SVC, IVC, PVs, and CS, CV *≈* 40 cm·s^−1^). The appropriate tissue conductivity value required to produce the desired CV for each region was determined using a 1D cable model with a mean element size similar to that of each respective region and is given in Table 4.

The 3D atrial geometry models were solved using the PARDISO finite element solver in COMSOL (v3.5a, COMSOL AB, Sweden). Quadratic Lagrange elements for the *E*_*m*_ variable and linear discontinuous elements for the gating variables were selected as options in the software. Approximately 3.5 hours were required to solve a 1-second simulation using a Dell Precision T7500 Workstation with x8 3.33 GHz CPUs and 126 GB of RAM running Windows XP x64 bit using a relative tolerance of 1.0 × 10^−4^, absolute tolerance of 1.0 × 10^−3^, and a maximum time step of 0.2 ms. 

## 3. Results

### 3.1. Optimisation of cSAN-pSAN-Atrial Disc

Spontaneous APs were recorded from cSAN, pSAN, and RA intact myocytes from the same tissue preparation. Although the APs were recorded from three tissue-intact myocytes in the same preparation, there were slight differences in the cycle length (CL) of cSAN, pSAN, and RA APs —possibly due to changes in the tissue's electrophysiological properties over time. To achieve identical CLs, modified pSAN and RA AP traces were reconstructed by pacing the optimised pSAN and RA single-cell models at a CL that matched the spontaneous CL of the recorded cSAN intact myocytes. 

**Figure 6:**
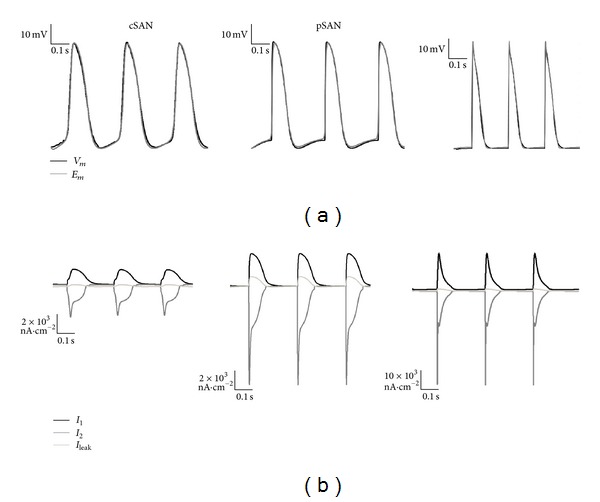
Optimised cSAN and pSAN models using a cSAN-pSAN 2D tissue axisymmetric disc model with *σ*_SAN_ = 800 *μ*S·cm^−1^. (a) The optimised cSAN and pSAN AP waveforms (*E*_*m*_) are overlaid on top of AP traces recorded experimentally (*V*_*m*_) from cSAN, pSAN, and RA myocytes, respectively, from a rabbit sino-atrial tissue preparation. (b) Inward, outward, and leakage ionic currents reconstructed from the optimised cSAN, pSAN, and RA models, respectively. Note the different scales used for currents in cSAN, pSAN, and RA.

A generic ionic model with two time-dependent (inward and outward) currents and one background current was implemented for all cell types in the 2D axisymmetric disc.  Figure 6 illustrates the optimised model and experimental AP waveforms for cSAN, pSAN, and RA cells for SAN tissue conductivity (*σ*_SAN_) of 800 *μ*S·cm^−1^ and atrial tissue conductivity (*σ*_RA_) of 10^4^ *μ*S·cm^−1^.  Table 3 lists the optimised model parameters for all cell types. The root mean square error (RMSE) between experiment and model APs was 2.22 mV, 2.45 mV, and 4.12 mV for cSAN, pSAN, and RA cells, respectively. There was a transition in the reconstructed currents from cSAN to RA models, with the peak magnitude of each time-dependent current increasing from cSAN to pSAN to RA (Figure 6). The SAN activated spontaneously and was able to entrain the RA region. AP propagation velocity was 22 and 96 cm·s^−1^ in the SAN and RA segments, respectively. The maximum rates of change of the membrane potential during phase 0 depolarisation and phase 1 repolarisation of the tissue-optimised RA AP were 46.03 and 1.689 V·s^−1^, respectively, compared to 26.55 and 0.7114 V·s^−1^ for the single-cell optimised RA AP.

### 3.2. Optimisation of LA-PV Disc

The parameters of the LA-PV disc were optimised to fit a series of three consecutive APs recorded intracellularly from the LA and PV in response to electrically pacing the tissue at a pacing interval of 400 ms. 

**Figure 7:**
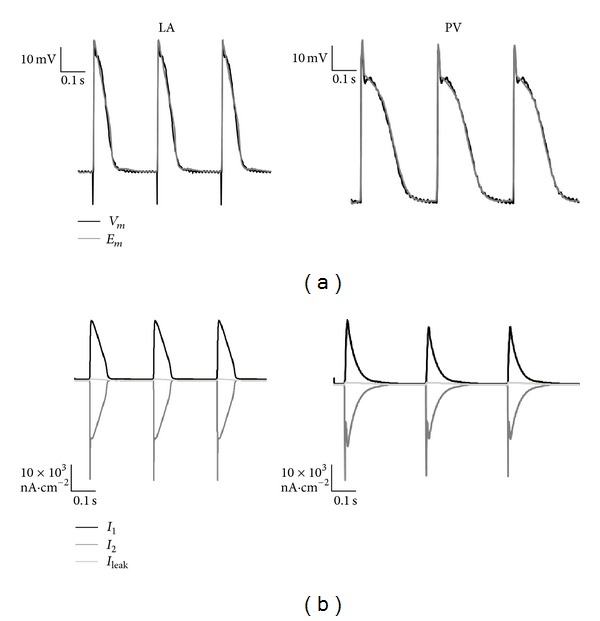
Optimised LA and PV ionic models using a combined LA-PV 2D axisymmetric disc model. (a) The optimised LA and PV AP waveforms (*E*_*m*_) are overlaid on top of AP traces recorded experimentally (*V*_*m*_) from LA and PV tissue-intact myocytes from respective rabbit tissue preparations. (b) Inward, outward, and leakage ionic currents reconstructed from the optimised LA and PV nodes in the combined disc model.

Figure 7 illustrates optimised and experimental AP waveforms for both LA and PV tissue-intact myocytes, and Table 3 lists the optimised model parameters for LA and PV cells in the combined PV-LA disc. The RMSE between experimental and model-generated AP waveforms was 3.92 mV and 2.38 mV for the LA and PV, respectively. The tissue conductivity of the entire disc was set to 10^4^ *μ*S·cm^−1^ producing an AP propagation velocity of 93 cm·s^−1^.

### 3.3. 3D Atrial Simulations

**Figure 8:**
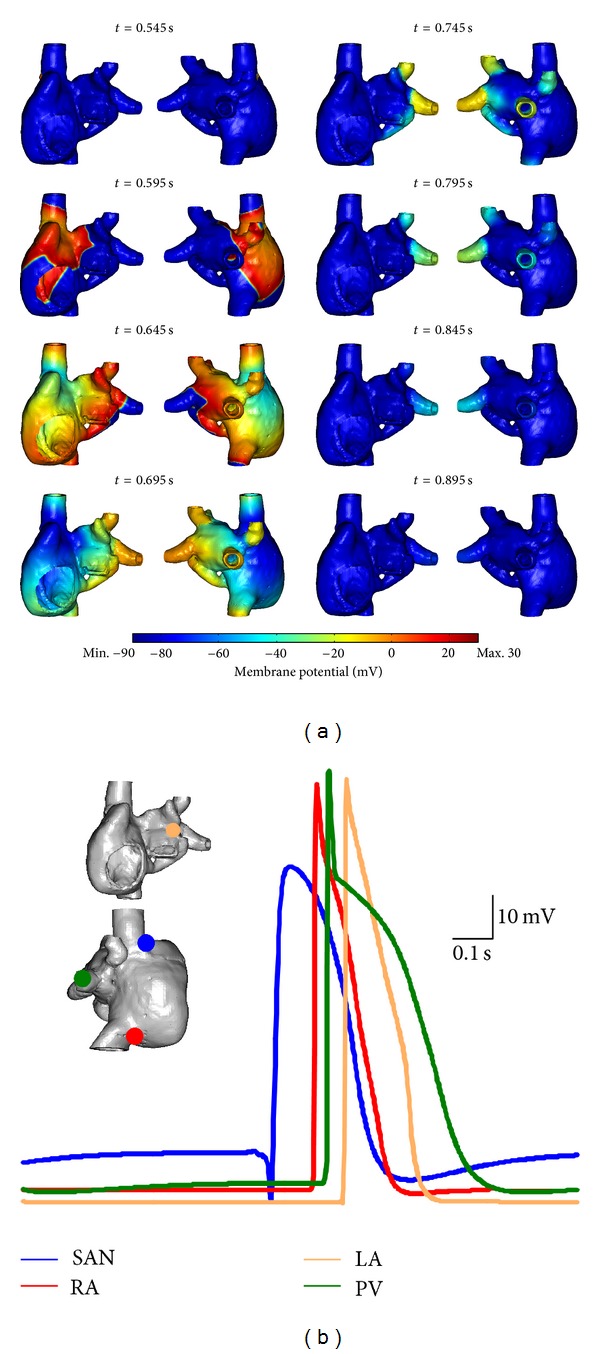
Rhythmic atrial simulation using optimised cSAN, pSAN, RA, LA, and PV ionic models and finite-thickness atrial wall geometry. (a) Snapshots of membrane potential across the surface of the atria at various times during one cardiac cycle. (b) Representative AP plots from various regions. The locations of points where the highlighted APs were sampled are shown in the inset. Data was sampled at 1 kHz.

In simulations utilising the 3D atrial geometry with finite wall thickness, spontaneous periodic APs originated from the SAN and propagated to activate the atria (Figure 8). APs in both the cSAN and pSAN regions were initiated at the same time and it took slightly longer for APs to propagate through the pSAN compared to the cSAN. Atrial breakthrough occurred first at the RA followed by the CT, with the activation pattern being stable for each cycle. Intra-atrial conduction began at the BB followed by the septum, with the CS playing only a minor role. The superior PVs were activated before the inferior ones. The mean pacemaking CL was 779 ms.

cSAN, pSAN, RA, LA, and PV APs were sampled from a random point in each respective region (Figure 8(b)), with AP waveforms exhibiting heterogeneous morphologies. In particular, the SAN AP displayed a prominent slowly depolarising pacemaker potential, which was absent in atrial tissue types. However a small slow pacemaking potential was present in the PV AP waveforms. The SAN exhibited a broad AP peak compared to the rapid phase 0 depolarisation and phase 1 repolarisation displayed by RA, LA, and PV APs. The cSAN possessed a more depolarised diastolic phase compared to the other regions.

**Figure 9:**
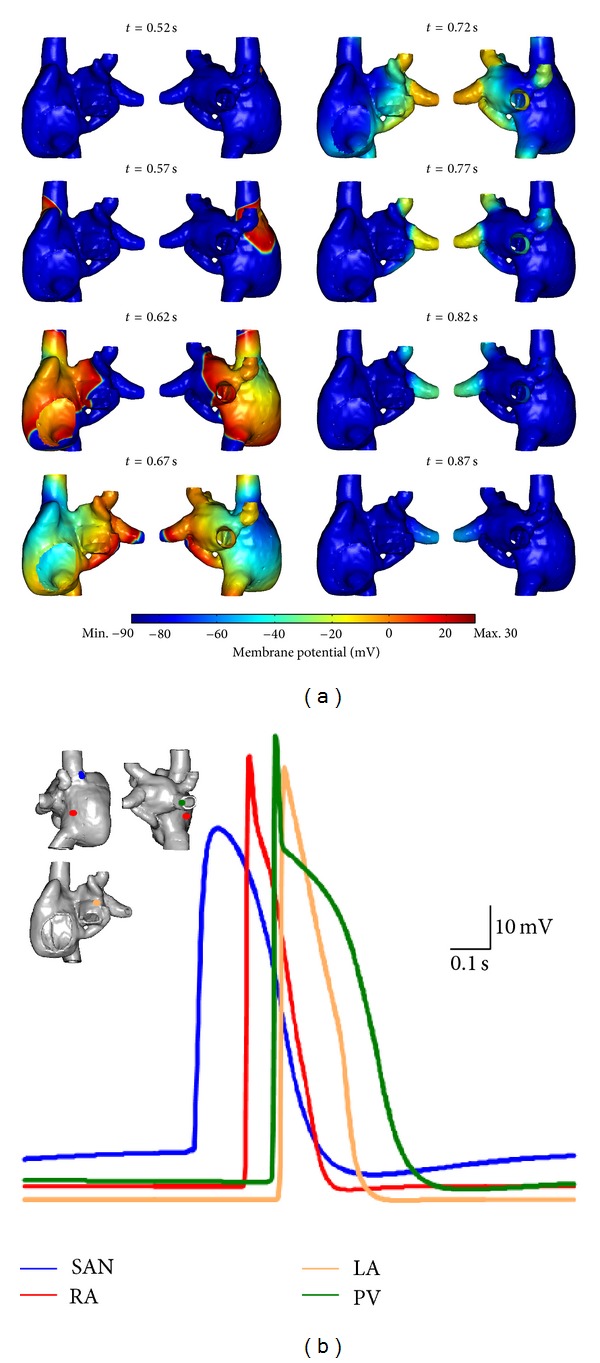
Rhythmic atrial simulation using optimised cSAN, pSAN, RA, LA, and PV ionic models and an atrial shell wall geometry. (a) Snapshots of membrane potential across the surface of the atria at various times during one cardiac cycle. (b) Representative AP plots from various regions. The locations of points where the highlighted APs were sampled are shown in the inset. Data was sampled at 1 kHz.

Simulations were also performed using a 3D atrial epicardial shell model (Figure 9). Spontaneous rhythmic APs also originated from the SAN and propagated to activate the atria. Initial activation occurred in the cSAN followed by the pSAN, and a longer time was required for the electric activation wavefront to propagate through the cSAN than the pSAN. Atrial breakthrough occurred first at the RA followed by the RAA and then the CT. The right PVs were activated first, followed by the left PVs. Pacemaking CL was 800 ms and the activation pattern across the aria was stable for all cardiac cycles. 

As in the case of the finite wall thickness simulation, AP waveforms sampled from various regions exhibited different morphologies (Figure 9(b)): in particular, the cSAN AP displayed a prominent slow pacemaker potential. The SAN exhibited a broad AP peak compared to the rapid phase 0 depolarisation and phase 1 repolarisation displayed in RA, LA and PV regions. The diastolic potential of cSAN was more depolarised than that of other AP types.

## 4. Discussion

A method has been developed which allows cardiac ionic models to be optimised to fit experimental AP waveforms recorded from *in vitro *tissue preparations (Figure 1). A generic cardiac ionic model was formulated, consisting of a user defined number of active time-dependent currents and a single leakage current. Each active current was described by two gating variables (*p* and *q*), each governed by voltage-dependent rates (*α* and *β*). By optimising parameters of the model, a variety of ionic currents could be reconstructed to produce morphologically distinct AP waveforms recorded from tissue-intact rabbit myocytes (cSAN, pSAN, RA, LA, and PV). To account for the presence of electrotonic coupling from neighbouring cells, 2D axisymmetric disc models with heterogeneous regions were optimised. These parameters were then imported into two models of 3D atrial geometry, reconstructed from cryosections of the male Visible Human, and able to simulate spontaneous SAN electrical activation and atrial propagation. Simulations were performed on both 3D shell and finite atrial wall thickness models. A monodomain formulation was used to describe electric propagation, with heterogeneous but isotropic tissue conductivity properties assigned to different regions to produce known CV values. This approach represents a move from single-cell model to tissue-based model optimisation, in an attempt to bridge the gap between cellular, tissue, and whole-organ multiscale cardiac electric models. Such a model of electrotonically interacting heterogeneous regions can provide valuable insights into the initiation and maintenance of atrial arrhythmias, as well as their possible treatment by pharmacological or ablation therapies.

Although in this study the modelling process was applied to atrial electrophysiology, the basic methodology outlined can be utilised to optimise any biological system model, in particular excitable cells and tissue. For example, a morphologically realistic model of a single neuron, with distinct model parameters for the dendrites, soma and axon, or even a network of such neurons, can be built by first optimising simplified multicompartmental models of heterogeneous neuronal segments. Similarly the modelling process can be used to optimise models of the interaction of neurons and either skeletal or smooth muscle at neuroeffector junctions. 

Methodologies for development of image-based models of cardiac electrophysiology have been proposed and applied to construct models of rabbit [24] and porcine [25] ventricles under healthy and pathophysiological conditions. Vadakkumpadan et al. have utilised diffuse tensor MR images coupled with the biophysically detailed Mahajan-Shiferaw [26] rabbit ventricular ionic model [24]. Such models offer high temporal and spatial resolution at the drawback of being computationally expensive [27]. On the other hand, Pop et al. have opted to use a phenomenological model in a geometry also reconstructed from MRI data [25]. The Aliev and Panfilov model [28] was optimised to reproduce the AP duration and CV recorded *ex vivo* in the ventricles by means of optical imaging [25]. This approach offers improved computational efficiency and a match between model and simulation activation patterns but a phenomenological model is unlikely to describe complex changes that occur in the AP morphology during fibrillation and cannot shed insights on the underlying ionic currents. In this study, a generic ionic model was chosen with standard template equations for ionic currents and gating kinetics. The modular nature of the model allows the user to specify the number of time-dependent ionic currents. Another advantageous feature of the proposed modelling process is the automated refinement of the model parameters using a custom curvilinear gradient optimisation algorithm to enable the generation of a variety of AP waveforms regardless of the complexity of the experimental conditions under which they were recorded. For purposes of computational efficiency, we have attempted to use the simplest model structure possible to reproduce experimental AP data. The model presented in this paper utilising two active currents and a leakage current is necessarily a simplification of the many ion channels likely present in the membrane. *i*_1_ and *i*_2_ here would likely represent the combination of multiple inward and outward currents carried by several ion species. Prior to optimisation, we make no assumptions as to the ionic identity of each current, but allow the fitting process to determine the current density and kinetics based solely on the AP data. Additional currents can be added into the model to provide more degrees of freedom needed to fit more complex experimental data. Moreover, a number of generic currents can be combined to reproduce the behaviour of complex currents such as *I*_*k*1_ or calcium transmembrane currents and intracellular dynamics. The curvilinear gradient optimisation method is robust enough to reproduce any given experimental data given the appropriate number of generic currents is used and sufficient computational resources are available. This approach represents a compromise between biophysically detailed and simplified phenomenological models. 

### 4.1. 2D Tissue Disc Model Optimisation

Cardiac tissue behaves as an electrical syncytium, in which the cells are electrically connected via gap junctions. An intracellular potential difference between neighbouring cells will produce an electrotonic coupling current affecting the dynamics of gating and rate variables in each cell, altering the overall AP morphology. Such an electrotonic current is not typically taken into consideration when formulating single-cell ionic models. However, single-cell model behaviour under electrotonic loading from other cell types is expected to change and may not produce physiological responses. For example, earlier studies using single-cell parameters for cSAN and pSAN ionic models had to be manually [17] or automatically [29] adjusted in order to produce sino-atrial activation in tissue stimulations. Alternatively, it is possible to adjust tissue conductivity and ion channel density gradients to avoid suppression of the SAN by atrial electrotonic loading or exit block from the SAN to the atrium as was implemented in a 3D right atrial model examining autonomic regulation of the cardiac pacemaker [19] using Zhang's equations [18] to model the SAN. Even if a single-cell model can be optimised to fit those experimental AP waveforms perfectly, it is important to note that the single-cell parameters are also producing unrealistic underlying ionic current behaviours that are compensating for the electrotonic coupling current that is present under experimental conditions but lacking in the model. 

Therefore, when optimising models to fit APs recorded from tissue preparations, it is important to use tissue-based models, however simplified, to reproduce the electrotonic interactions. Dastgheib et al. [16] were the first to report the use of a 1D ring model in parameter optimisation and proposed that such a setup was better able to simulate electrotonic coupling, physiological excitation and propagation. However, only parameters describing the maximum conductance of ion channels were optimised and none related to channel kinetics. We used a generic ionic cardiac model to construct a 1D cable equivalent of an axisymmetric 2D tissue disc consisting of one or more segments representing different cardiac regions. Although only two time-dependent and one background currents were used, the 2D discs were able to produce the transition in AP morphology and underlying ionic currents from SAN to RA, and LA to PV. In addition the rates of membrane potential change during phase 0 depolarisation and phase 1 repolarisation were greater than those obtained during single-cell optimisation and better matched experimental observations. The results from this study are consistent with the proposal of Syed et al. that the use of physiologically realistic stimulus currents to evoke APs during optimisation will result in improved fits to tissue AP waveforms [11], especially during the initial AP phases where the electrotonic current contribution to the AP is most significant. This study is an improvement on the approach of using the second time derivative of *V*_*m*_ as an estimate of the electrotonic “stimulus” current [11] as the use of a cable equation produces a more accurate description of the electrotonic current needed to bring a given cell's membrane potential to threshold to fire an AP, particularly in the presence of electrically heterogeneous tissue regions. 

### 4.2. 3D Atrial Models

The atrial geometry included the SAN, RA, RAA, CT, LA, LAA, interatrial tissue (septum, BB, and CS), both SVC and IVC, and four PVs and openings for the tricuspid and mitral valves, but no pectinate muscle fibres, as present in the Harrild and Henriquez [3], Seemann et al. [4], and Aslanidi et al. [6] atrial models. The 3D finite atrial wall thickness model improves on the Harrild and Henriquez [3] and Aslanidi et al. [6] atrial models in that it includes the PVs. It also improves on the Seemann [4] and Aslanidi et al. [6] atrial geometries by incorporation of the CS. Preliminary simulations [30] and previous studies [4, 31] suggest that the CS plays a role during reentry. An epicardial shell surface model was also derived from the atrial geometry. The shell surface incorporated the cSAN, pSAN, PVs, SVC, IVC, and the CT. These structures were absent in the Virag et al. [5] shell, which was used extensively to simulate AF and ablation therapy in previous modelling studies (e.g., [5, 32] although the atrial shell of this study did not have a distinct septal sheet with a fossa ovalis, as utilised in Virag et al. [5]).

In both versions of our atrial model, the SAN overcame the electrotonic load imposed by the surrounding atrial tissue and was spontaneously active, rather than being externally paced (e.g., [3, 5]). The SAN was able to generate rhythmic APs and activation wavefronts that propagated into and excited the atria. Unlike Seemann et al. [4] who used numerical interpolation to divide the SAN into central and peripheral regions, we explicitly divided the SAN geometrically, ascribing to each part separate ionic parameters. 

Mesh element sizes for the finite wall thickness and epicardial shell models (3.388 and 0.991 mm, resp.) were larger than the mean element sizes typically reported for atrial meshes (e.g., 550 *μ*m [3] and 600 *μ*m [5]) or, in the case of the finite wall thickness model, the space constant (0.5–2 cm) of cardiac tissue [33]. The imaging resolution of the male Visible Human cryosection dataset did not allow for the discrimination of the microstructure of the SAN and its anatomical connection to the surrounding atrium. Hence in the finite-thickness version of the model, spatial resolution is not resolved enough to capture propagation from the leading pacemaker site in the SAN to the SAN periphery. The epicardial model utilises a boundary shell mesh with a finer resolution and hence is able to simulate cSAN activation followed by pSAN and then atrial excitation. A future improvement of the model would be selective refinement of the SAN domains using images obtained from immunohistochemical studies or high-resolution MRI scans of the atria. A mesh size validation study using representative geometries was conducted and only a 10% change in atrial CV was found when the mean element size was reduced to 246 *μ*m. In addition, the size of the mesh elements was compensated for by calculating the appropriate tissue conductivity value to produce the required CV for each particular region, an approach also adopted by Laurent et al. [34]. In addition, quadratic, rather than linear, Lagrange elements were used numerically to solve for the *E*_*m*_ variable in the finite element models, which will further improve the accuracy of the solution. Moreover, the simulations presented in this manuscript are of regular rhythmic sinus activation patterns and not of arrhythmia so the large element size will not have a significant effect on the solutions presented in this paper.

One limitation of the atrial models of this study was that the ionic single-cell models from which they were constructed were based on fits to rabbit APs, whereas the 3D atrial geometry was based on human data. Nonetheless, the generic ionic model approach could readily be applied to human atrial AP data if this was available and incorporated into patient-specific reconstructions of atrial geometry. This would greatly improve the accuracy of the model. Another limitation is that our geometry does not include myocardial fibre or pectinate muscle orientation which is known to play an important role in atrial conduction ([35], also refer to a recent atrial sheep model for a discussion on the role of atrial myoarchitecture in electric propagation [36]). Unfortunately, this data was not available from the low-resolution cryosection images used but would also greatly improve model accuracy if included. Furthermore, tissue conductivity in both disc and 3D models was set empirically to obtain realistic AP conduction velocities in each region, taking into account the spatial discretisation and mesh element size. A range of values for myocardial tissue conductivity have been reported in existing modelling studies [3–6] and are typically assigned to yield realistic conduction velocities of about 50–150 cm/s [37]. Conduction velocity is determined by the combination of membrane sodium channel density and kinetics, as well as the gap junction density between myocytes. The latter is described using the tissue conductivity value. The former depends on the membrane conductance and gating kinetics of the inward Na^+^ current, which differs between ionic models. The ionic model of this study includes a generic inward current having particular kinetics and membrane conductance, which could explain the disparity between our tissue conductivity values and others reported in the literature. The model was fitted to AP morphologies recorded in response to a single pacing frequency as a demonstration of the feasibility of the proposed tissue-based modelling methodology. Fitting the generic model to more complex experimental waveforms that better capture the changes in AP morphology during fibrillation is required for more accurate 3D modelling of atrial arrhythmias. Towards this aim, the authors have recently developed [38] a single cell generic cardiac ionic model optimised to fit AP morphology alternans at a uniform pacing cycle length of 200 ms as well as the response to random pacing intervals. Using the tissue-based optimisation approach described in this paper, such ionic models can be incorporated into 3D atrial geometries to allow more realistic simulations of the dynamics of AF. Like most existing ionic models, our generic model has certain limitations [38], which are mainly due to assumptions made in order to simplify the model to produce computationally efficient simulations in 3D geometries as well as to optimise the disc models. We have not incorporated intracellular compartments for calcium cycling, ionic pumps and exchangers as we assume that all of our reconstructed membrane currents consist of two first-order voltage-dependent (*p* and *q*) gating processes, which is unlikely to capture the kinetics of these mechanisms. However this issue does not affect the successful application of the generic model in 3D simulations.

## 5. Conclusion

A modelling process was developed to fit cardiac ionic models to APs recorded experimentally from *in vitro *SAN and atrial tissue preparations using a heterogeneous tissue-based optimisation protocol. Optimised model parameters were incorporated into 3D atrial models to simulate electric activation patterns in the atria. Accurate modelling of electrophysiological properties based on fits to AP data is important in simulating complex atrial arrhythmias and their modulation by external pacing, pharmacological treatment or tissue ablation strategies. The tissue-based optimisation approach developed is a generic tool that can find broad applications in modelling subject or experiment-specific excitable tissue.

## Figures and Tables

**Table 1:** Constraints imposed on each parameter for each ionic model cell type during model optimisation.

Current	Parameter	cSAN		pSAN		RA, LA, PV	
		Lower limit	Upper limit	Lower limit	Upper limit	Lower limit	Upper limit
*I*_1_	$\overset{-}{g_{1}}$ (*μ*S·cm^−2^)	0	20000	10	10000	10	20000
	*E*_rev,1_ (mV)	−100	−70	−100	100	−100	−60
	*k*_*αp*1_ (s^−1^)	0	5000	0	5000	0	5000
	*s*_*αp*1_ (mV^−1^)	−5	0	−5	0	−0.2	0
	*k*_*βp*1_ (s^−1^)	0	5000	0	5000	0	5000
	*s*_*βp*1_ (mV^−1^)	0	5	0	5	0	0.2
	*E*_50,*p*1_ (mV)	−70	3	−80	20	−80	25
	*k*_*αq*1_ (s^−1^)	0	5000	0	5000	0	5000
	*s*_*αq*1_ (mV^−1^)	−5	0	−5	0	−0.2	0
	*k*_*βq*1_ (s^−1^)	0	5000	0	5000	0	5000
	*s*_*βq*1_ (mV^−1^)	0	5	0	5	0	0.2
	*E*_50,*q*1_ (mV)	−70	3	−100	100	−80	25

*I*_2_	$\overset{-}{g_{2}}$ (*μ*S·cm^−2^)	0	20000	10	10000	10	20000
	*E*_rev,2_ (mV)	5	100	−100	100	40	100
	*k*_*αp*2_ (s^−1^)	0	5000	0	5000	0	5000
	*s*_*αp*2_ (mV^−1^)	−5	0	−5	0	−0.2	0
	*k*_*βp*2_ (s^−1^)	0	5000	0	5000	0	5000
	*s*_*βp*2_ (mV^−1^)	0	5	0	5	0	0
	*E*_50,*p*2_ (mV)	−70	3	−80	20	−80	25
	*k*_*αq*2_ (s^−1^)	0	5000	0	5000	0	5000
	*s*_*αq*2_ (mV^−1^)	0	5	0	5	0	0.2
	*k*_*βq*2_ (s^−1^)	0	5000	0	5000	0	5000
	*s*_*βq*2_ (mV^−1^)	−5	0	−5	0	0.2	0
	*E*_50,*q*2_ (mV)	−70	3	−100	100	−80	25

*I*_*L*_	$\overset{-}{g_{L}}$ (*μ*S·cm^−2^)	0	20000	10	10000	10	20000
	*E*_rev,*L*_ (mV)	5	100	−100	100	−60	0

*I*_1_: outward time-dependent current, *I*_2_: inward time-dependent current, *I*_*L*_: leakage current.

**Table 2:** Initial values for the generic ionic model variables used in the 3D atrial models.

	cSAN	pSAN	RA	LA	PV
*E*_*m*_ (mV)	−70.26	−80.04	−78.74	−80.75	−78.79
*p*_1_	0.2	0.11	0.85	0.880	0.95
*q*_1_	1	1	0.5	0.3	0.072
*p*_2_	0	0	0	0	0
*q*_2_	0.36	0.35	0.89	0.29	0.78

**Table 3:** Optimised generic ionic model parameter values used in the 3D atrial models. Parameter values were obtained by optimising a cSAN-pSAN-RA and LA-PV 2D axisymmetric disc models.

Current	Parameter	cSAN	pSAN	RA	LA	PV
*I*_1_	$\overset{-}{g_{1}}$ (*µ*S·cm^−2^)	20.75	40.61	332.25	295.3	3098.3
	*E*_rev,1_ (mV)	−83.96	−97.57	−86.46	−93.4	−91.9
	*k*_*αp*1_ (s^−1^)	4775.9	3202.9	2008.02	4805.4	1415.6
	*s*_*αp*1_ (mV^−1^)	−3.56	−0.72	−0.158	−0.094	−0.087
	*k*_*βp*1_ (s^−1^)	16.02	50.02	75.02	0	4
	*s*_*βp*1_ (mV^−1^)	3.11	0	0	0.058	0.002
	*E*_50,*p*1_ (mV)	−60.71	−53.04	−63.97	−76.5	−70.4
	*k*_*αq*1_ (s^−1^)	14.22	142.89	198.22	327	18.4
	*s*_*αq*1_ (mV^−1^)	−5	−1.99	0	−0.054	−0.001
	*k*_*βq*1_ (s^−1^)	0.21	58.92	293.23	2041.5	1198.3
	*s*_*βq*1_ (mV^−1^)	0.16	0.76	0.17	0.06	0.2
	*E*_50,*q*1_ (mV)	−21.86	−97.73	−0.83	−79.9	−11.5

*I*_2_	$\overset{-}{g_{2}}$ (*µ*S·cm^−2^)	6758.38	2856.22	6904.07	4479.9	16283.6
	*E*_rev,2_ (mV)	16.19	8.12	40.01	43.5	40
	*k*_*αp*2_ (s^−1^)	4.37	123.81	562.54	2000	241.3
	*s*_*αp*2_ (mV^−1^)	−3.7	−4.19	−0.185	−0.198	−0.2
	*k*_*βp*2_ (s^−1^)	3347.16	2643.18	4999.1	1005.4	4770.6
	*s*_*βp*2_ (mV^−1^)	0.14	0.06	0.001542	0.058	0.001
	*E*_50,*p*2_ (mV)	−62.77	−67.13	−44.12	−42.2	−43.5
	*k*_*αq*2_ (s^−1^)	3.68	4.86	30.97	10	10
	*s*_*αq*2_ (mV^−1^)	3.32	1.62	0.123	0.199	0.122
	*k*_*βq*2_ (s^−1^)	35.32	28.56	44.85	43	27.7
	*s*_*βq*2_ (mV^−1^)	−4.99	−1.85	−0.18	−0.004	−0.159
	*E*_50,*q*2_ (mV)	−48.39	−69.48	−60.15	−63.3	−64.8

*I*_*L*_	$\overset{-}{g_{L}}$ (*µ*S·cm^−2^)	2.93	16.047	13.5	10	11.5
	*E*_rev,*L*_ (mV)	5.07	−71.67	−57.5	−51.9	−60

*I*_1_: outward current, *I*_2_: inward time-dependent current, *I*_*L*_: leakage current.

**Table 4:** Tissue conductivity and surface to volume values used in the 3D atrial models.

	Region	Epicardial shell model	Finite thickness atrial wall model
*σ* (*μ*S·cm^−1^)	cSAN	5 × 10^2^	5 × 10^2^
	pSAN	5 × 10^2^	5 × 10^2^
	RA	1.41 × 10^4^	1.28 × 10^4^
	RAA	1.41 × 10^4^	1.28 × 10^4^
	Septum	1.41 × 10^4^	1.28 × 10^4^
	LA	1.04 × 10^4^	5.01 × 10^4^
	LAA	1.04 × 10^4^	5.01 × 10^4^
	BB	2.77 × 10^4^	2.13 × 10^4^
	CT	4.05 × 10^4^	4.11 × 10^4^
	SVC-IVC	2.45 × 10^3^	1.28 × 10^3^
	CS	2.45 × 10^3^	1.28 × 10^3^
	PV	1.58 × 10^4^	1.36 × 10^4^

*A* (mm^−1^)	SAN	500	500
	Other regions	200	200
